# A charter for biomedical research ethics in a progressive, caring society

**DOI:** 10.1186/s13010-015-0032-y

**Published:** 2015-11-13

**Authors:** Sylvie Vandoolaeghe, Alessandra Blaizot, Danie Boudiguet, Valérie Bougault, Eduardo Dei Cas, Benoît Foligne, Anne Goffard, Hélène Lefranc, Bénédicte Oxombre, Thomas Trentesaux, Bernard Vandenbunder, Isabelle Wolowczuk, Laurence Delhaes, the “ethic and research” working group

**Affiliations:** Departement of Ethics, Espace Ethique Hospitalier et Universitaire de Lille (EEHU), Lille University Hospital, Faculty of Medicine, F-59000 Lille, France; University of Lille, F-59000 Lille, France; School of Dentistry, Lille University, F-59000 Lille, France; University of Paris Descartes Laboratory of Medical Ethics and Forensic Medicine, EA 4569, F-75008 Paris, France; School of Anaesthetist Nursing, Lille University Hospital, F-59000 Lille, France; EA 4488 “Activité Physique, Muscle, Santé”, Faculty of Sports Sciences, F-59790 Ronchin, France; Department of Microbiology, Lille University Hospital, Faculty of Medicine, F-59000 Lille, France; Biology and Diversity of Emerging Eukaryotic Pathogens Research Group (BDEEP), F-59019 Lille, France; Center for Infection and Immunity of Lille (CIIL), Institut Pasteur de Lille (IPL), F-59019 Lille, France; UMR CNRS 8402, F-59021 Lille, France; CIIL, IPL, Lactic Acid Bacteria and Mucosal Immunity Research Group, Center for Infection and Immunity of Lille (CIIL), Institut Pasteur de Lille (IPL), F-59019 Lille, France; University Lille, CNRS, Inserm, CHU Lille, Institut Pasteur de Lille, U1019 - UMR 8204 - CIIL - Centre d’Infection et d’Immunité de Lille, F-59000 Lille, France; Délégation à la Recherche Clinique et à l’Innovation, Maison Régionale de la Recherche Clinique Hospitalière et Universitaire, Lille University Hospital, F-59037 Lille, France; LIRIC - UMR 995, F-59000 Lille, France; Interdisciplinary Research Institute, USR 3078 CNRS, University of Lille 1, F-59658 Villeneuve d’Ascq, France; Lung Infection and Innate Immunity (LI3), BP 245, F-59019 Lille, France

**Keywords:** Charter, Ethics, Research, Life science, Integrity

## Abstract

**Background:**

Given that advances in research continuously raise new ethical issues, a multidisciplinary working group of investigators involved in biomedical research has gathered to discuss and compare ethical viewpoints in their daily practice.

**Methods:**

The working group has drafted a Charter for Ethics in Biomedical Research that encompasses all the steps in the research process, i.e. from the initial idea to analysis and publication of the results.

**Results:**

Based on key principles for ethically responsible research, the Charter may serve as a tool for performing research, discussing research issues and training researchers.

**Conclusions:**

The Charter should stimulate researchers to think about their responsibility for research in a progressive, caring society.

## Introduction

Over the past few decades, outstanding progress in the field of biotechnology has increased awareness of ethical issues. Researchers, national bodies and international organizations have therefore been prompted to develop guidelines on research ethics and integrity [1].

A well-known chapter in the history of research with human subjects opened after World War II, with the establishment of the Nuremberg Code [2]. This was followed by the Declaration of Helsinki, which constitutes the basis for all subsequent national and international ethical guidelines [3]. However, the ethical concepts in these important international guidelines do not cover aspects of daily research practice. In the field of biotechnology, a range of questions concern aspects of a researcher’s day-to-day work: training, practice, societal and professional roles, objectivity, credibility, evaluation and constraints. In 1983, France was the first country to create a National Consultative Committee on Ethics in the Health and Life Sciences (Comité Consultatif National d'Ethique *pour les sciences de la vie et de la santé*, CCNE) to “provide advice on ethical issues and social issues raised by the progress of knowledge in the fields of biology, medicine and health”. Soon after the foundation of the CCNE, France introduced legislation on the creation of ethics forums and “Think Tanks” in French hospitals. Hence, Lille University Hospital and the Lille Faculty of Medicine launched the *Espace Ethique Hospitalier et Universitaire de Lille* (EEHU; http://www.eehu-lille.fr/) in 2010. Since 2012, the “Ethics and Research” working group has met within the framework of the EEHU (under the impetus of Dr Eduardo Dei Cas [4–6]) to consider the role of life and health science researchers in a university hospital in the 21st century (from fundamental research to clinical applications). After a number of multidisciplinary debates on issues generated by the research, progress in research and the representations of research that ensue, the group came to feel that the fruit of its discussions could be usefully summed up as a guide to ethical issues for researchers. The present Charter for Biomedical Research Ethics in a Progressive, Caring Society is intended to be simple, open to challenge and revision, and accessible to all researchers and stakeholders involved in health and life science research. The Charter provides a number of practical tools for researchers:

- A tool for improving research and ethical practices: Enhancing the quality and legitimacy of research, emphasizing the researcher’s individual responsibility and promoting personal consideration and collective debate on research ethics.
- A tool for debate and dialogue within research groups: Facilitating interactions within the research group, between research groups, and between research groups and institutions.
- A training tool for students and young researchers: Facilitating learning and counselling, encouraging the proper supervision of young researchers, fostering their awareness of ethics and introducing them to research ethics.

## Materials and methods

### Constitution of an “Ethics and Research” working group

In 2011, individual and institutional stakeholders in the life and health sciences based in and around the city of Lille (northern France) were invited to form a working group. Notably, people with expertise in medicine, pharmacy, dentistry, nursing, sports science, biology, chemistry, physics, literature, philosophy and languages were invited to participate. Ultimately, a group of 14 people from a wide range of areas of biomedical research met to discuss research ethics (Table 1). The group meets every two months to discuss issues and share its members’ experiences. It combines critical thinking and ethical analysis within a framework of discourse ethics [7]. The group’s mission goes beyond normative ethics, although it does question the sources, grounds, and legitimacy of ethical matters.

**Table 1:** The members of the multidisciplinary “Ethics and Research” working group

First name and family name	Qualifications/position/field of research	Affiliation
Alessandra Blaizot	DDS, MSc/Ethics	CHRU^a^ of Lille, University Hospital, University of Lille
Danie Boudiguet	Director of Nursing, Nurse anaesthetist (I.A.D.E^b^ school) RNA/Teaching and training	CHRU of Lille, University Hospital
Valérie Bougault	PhD/Physiology & Sport sciences	University of Lille
Eduardo Dei Cas	MD, PhD/Mycology & Parasitology	CHRU of Lille, University Hospital, University of Lille
Laurence Delhaes	MD, PhD/Mycology & Parasitology	CHRU of Lille, University Hospital, University of Lille
Benoît Foligné	PhD/Microbiology & Immunology	Institut Pasteur de Lille, University of Lille
Anne Goffard	MD, PhD/Virology	CHRU of Lille, University of Lille
Hélène Lefranc	BSc, Hospital Engineer at DRCI^c,^, Clinical Research Project Manager/Research Management	CHRU of Lille University Hospital
Bénédicte Oxombre-Vanteghem	PhD/Neuro-immunology	University of Lille
Thomas Trentesaux	DDS, PhD/Ethics	CHRU of Lille, University Hospital, University of Lille
Bernard Vandenbunder Vandenbunder	PhD/Physics - Cell Biology - Biophotonics	Centre National de la Recherche Scientifique
Sylvie Vandoolaeghe	MSc, Assistant Coordinator of the Lille EEHU^$$^/Philosophy	CHRU of Lille, University Hospital
Isabelle Wolowczuk	PhD/Immunology and metabolism	Centre National de la Recherche Scientifique

^a^Centre Hospitalier Régional et Universitaire. ^b^Infirmier Anesthésiste Diplômé d’Etat. ^c^Délégation à la Recherche Clinique et à l’Innovation. ^$$^ *RNA* Registered Nurse Anaesthetist, Centre National de la Recherche Scientifique. *EEHU* Espace Ethique Hospitalier et Universitaire

### Methods

The “Ethics and Research” working group searched electronic databases and the Internet to identify articles, reviews, editorials, books or any related content in academic journals and/or on websites that refer to research ethics, a charter for ethics in biomedical research or research integrity. The analysis included international charters (such as the Singapore Statement on Research Integrity), national charters (such as those established in Belgium and Sri Lanka) and ethical charters drafted by learned societies [2, 8–10].

## Results

### The charter for biomedical research ethics

The Charter for Biomedical Research Ethics is based on debate, open-mindedness and dialogue [4–6, 8–12]. Rather than imposing additional constraints on researchers, the Charter’s objective is to put forward common points of reference and principles for action for those working in the fields of life and health science.

The Charter is based on the following principles:

- Integrity, honesty, impartiality, transparency and critical thinking.
- Competence, accuracy, rigorousness, precision and verifiability.
- Caution, carefulness, respect, confidentiality and reliability.
- Independence of mind, creativity, open-mindedness and altruism.

The Charter describes how these principles can be translated into actions during the different steps in the research process, from the initial working hypothesis to publication and dissemination of the results and consideration of opportunities for further research. The Charter addresses the following five aspects of research:

### Defining the context and objectives of research

As an essential prerequisite to a research project, the researcher must determine the “state of the art”. This enables her/him to (i) present and acknowledge the field’s results and current limitations (*integrity and honesty*), (ii) cite the corresponding authors, and (iii) think of ways of advancing the state of the art (*critical thinking and open-mindedness*).

The researcher should precisely define her/his working hypothesis by delimiting the field of investigation *(rigorousness)*. She/he should take account of what is known from previous research, while seeking to add value. Accordingly, she/he will justify her/his efforts to make further progress *(creativity and altruism)*.

### Selecting materials and setting up methods

#### Methodology (integrity, rigorousness and verifiability)

The researcher should select the methodology that is best suited to answering the research question. Methods should not be chosen or adapted in order to obtain pre-defined results. The researcher must check that the chosen materials and methods are appropriate and can be used optimally under the research project’s various constraints. She/he must seek to reduce experimental bias as much as possible, while considering the limitations of her/his approach.

From the beginning of the work onwards, all raw data should be stored so as to ensure rigorousness and transparency in accordance with current legislation. This requirement covers the research hypothesis, communication with various organizations or journals, bibliographic references, the methods used, the data collected, and the structuring, analysis, publication and communication of results. Compliance with these criteria helps to structure the research and enables research processes to be verified and replicated.

### Commitments, interests and working relationships

The researcher must not overrate her/his work or mislead funding bodies, research institutions, colleagues, the media or the general public *(integrity, honesty, and accuracy)*.

The researcher must state the identity of her/his sponsors and funding sources and must describe the nature of relationship(s) she/he has with them (e.g. acting as a consultant or an expert advisor). She/he must declare any personal, professional or financial interests in order to maintain the necessary level of trust in her/his own research and (as an extended responsibility) in research in general. In the event of a potential conflict of interest, the researcher can only agree to participate if her/his impartiality is beyond doubt.

When a researcher is asked to engage in a research activity, she/he must always honestly estimate her/his ability to successfully complete that activity. She/he should commit to maintaining and upgrading her/his skill level and knowledge and to achieving proficiency in the techniques required to advance her/his research *(competence)*.

The researcher must carry out her/his research in accordance with the current legislation and deontological and institutional guidelines, rules and regulations *(legality)*.

She/he must respect the colleagues and students which whom she/he works by treating them fairly and without subjecting them to discrimination or any kind of abuse *(respect, courtesy and loyalty in working relationships within the group, between groups, towards her/his colleagues and hierarchy)*. She/he should seek to manage tensions within a group as best she/he can and must acknowledge the constraints related to employment contracts.

### Participants (caution, carefulness, respect, confidentiality and reliability)

The researcher and her/his institution are ethically obliged to take account of the risk-benefit ratio associated with research work. In clinical research, the “subjects” are human beings and so their dignity, freedom and privacy must be protected. The researcher must minimize the risk of harm to others, and should act with caution and foresight. She/he should take specific precautions to protect vulnerable persons. When the research is performed in a country other than her/his own, she/he should respect the local culture and environment. She/he should admit to and correct (to the greatest extent possible) any mistakes, omissions or damage caused. Moreover, the researcher must be cautious and take precautions if her/his experiments are likely to have an environmental impact.

For biomedical research involving human subjects, the sponsor must obtain approval from the relevant independent ethics committee/investigational review board.

If animals are used, the researcher must treat them carefully and respectfully. She/he must limit the animals’ suffering, in line with the “3Rs” (Reduction, Replacement, and Refinement). She/he must not implement an unnecessary or poorly designed animal experiment or any animal experiment that has not been approved by the relevant institutional animal care and use committee.

### Analysing results

When analysing research results, the researcher must not disclose confidential personal data (*respect, carefulness and confidentiality*). The researcher should not be let her/his preconceived ideas influence the analysis or interpretation of her/his results. She/he should differentiate between her/his own opinions and the scientific experiment (*impartiality*). She/he must also distinguish between hypotheses and speculations on one hand and the results and conclusions of her/his research on the other (*accuracy*). The researcher should avoid bias when analysing and interpreting her/his own data. According, peer reviews or reviews of a colleague’s work should be objective (*integrity and objectivity*).

The researcher must seek to avoid negligence (*rigorousness*). She/he should maintain a critical view of her/his own work and that of her/his peers - particularly during collaborative work (*critical thinking*). In interdisciplinary projects, she/he should place the requisite trust in colleagues and pay attention to the different views that they may bring to her/his area of expertise (*open-mindedness*).

A researcher’s analysis of the results and her/his subsequent establishment of the conclusions must be driven by a requirement for truth and knowledge. The research must not be transferred too quickly to the media or business stakeholders outside the scientific community. These results must not be falsified by constraints, obligations or pressure. In contrast, results should not be set aside for too long in an effort to “protect” them.

When the researcher evaluates the research of her/his peers, she/he should carry out the mission thoroughly, fairly and without disclosing confidential information. She/he shall review papers and grant applications in a timely manner and from a neutral viewpoint, and must be guided solely by scientific considerations. Reviewing is an important part of her/his overall responsibility to the discipline of research. When supervising students, she/he shall offer appropriate advice and promote competence and responsibility (*responsible tutoring*).

### Communicating and publishing research results

The researcher must comply with patent rights, copyright and other intellectual property rights. She/he acknowledges other people’s work and contributions. She/he must not present other people’s work as her/his own. She/he must respect her/his colleagues’ ideas and never plagiarize them [8] (*honesty and accuracy*).

The researcher accepts responsibility for her/his contributions as a group member, co-author or author. When publishing, the author should honestly distinguish between (i) co-authors who meet the criteria for authorship and (ii) colleagues who should solely be acknowledged (i.e. minor contributors).

The principal investigator should appropriately manage her/his group members’ respective contributions by precisely defining their respective roles (*rigorousness).*

The researcher should communicate her/his results in a timely and open manner, in order to establish ownership of intellectual property and establish priority. Raw data must be stored, so that they can be validated and/or replicated (*transparency*, *rigorousness*, *precision and verifiability*). The researcher takes account of all her/his results - including unwanted or unexpected ones – and should not filter them arbitrarily (*accuracy*). She/he should not disclose confidential information on patients, participants or other people involved in research before, during or after publication of the results (*confidentiality*). However, on request, the results should be made available to the participants.

### Defining follow-up actions and perspectives for further research

By performing high-quality research with integrity, the researcher creates and maintains confidence in her/his work. In this respect, she/he must be alert to any lack of integrity within her/his research group (*responsibility*).

In order to enable and promote responsible behaviour by the researcher, her/his research environment should:

- promote competence via access to continuing education and training.
- set clear, realistic working rules that are compatible with the research project’s time-frame and constraints.
- implement appropriate actions to correct or retract publications, if required.
- define fair criteria for career advancement and thus enable both individual and collective development.

## Discussion

Scientific research is rooted in the quest for truths and knowledge that serve humanity; it fits into a vision of a progressive, caring society. Researchers must have freedom of speech and intellectual independence with regard to their science, from hypotheses through to implementation and conclusions. In this respect, funding agreements are drawn up to precisely define the rights and obligations of each party (including those of the research institution) and to manage intellectual property, communication and exploitation of the results. However, the research process must always leave some room for serendipity.

Given that (i) today’s societies have many points of reference, (ii) scientific and biomedical practices are constantly evolving and (iii) the use of scientific and biomedical practices may create dilemmas and raise obstacles, we need to find a way of thinking about research ethics that enables us to question the legitimacy of the means employed and the research’s possible consequences. This approach is necessarily placed in a specific context - here and now, with circumstances, constraints, a past and a future. The present Charter for Biomedical Research Ethics is not a tool for accrediting a given research project as being "ethical" or not; in fact, it incites each researcher to think about the objectives she/he defines, the means she/he uses to achieve them, and the risks and limitations related to her/his research. Accordingly, the researcher takes responsibility not only for her/his own research but also for research in general.

Research in the life and health sciences generates representations of the living world [12] via (a) frameworks for conceptualizing research hypotheses, (b) biomedical practices (in which a patient is considered as an object, e.g. in biotechnology), (c) policies on precaution and prevention, (d) education and training [13, 14], (e) a rationale for political decisions on biological research, (f) perceptions and images of the living world conveyed by the media and in the cultural and artistic domains.

Biomedical research deals with emergence of the complexity of the living world in its universality and singularity, which requires open-mindedness, critical thinking, multiple analytical strategies, creativity and unceasing conscientiousness. In view of the complexity and social impacts of research and its mutually stimulating disciplines (biomedical science, the human and social sciences, etc.), it is essential to question research’s resources, limitations and meaning. What are we aiming to achieve? What can be expected? What should be avoided? How can science and society move forward? As its title suggests, the present Charter for Ethics in Biomedical Research is primarily aimed at researchers in the fields of life and health sciences worldwide. However, it could be useful for other stakeholders in the paramedical professions and/or other fields of science. Indeed, when disciplines meet or are confronted with one another, a new vision of the living world *–* perhaps better adjusted to the complexity of life *–* may emerge. Along these lines, today’s research programmes are growing at the interfaces between nanoscience, computer science and biology. Some researchers recommend a global, integrated approach for strengthening the links between human health, animal health and environmental care (the “One Health” concept, for example [15]). Another example is the interdisciplinary workgroups (including representatives of the human and social sciences) organized by the North American “Global Health” consortium [16]. The European Union’s research directives for the “Horizon 2020” program [17] also encourage various types of multidisciplinary and/or international cooperation; these correspond to an innovative, globally coherent vision of issues that require international guidelines for research integrity [18].

In conclusion, we believe that present Charter for Ethics in Biomedical Research (based on common principles) will evolve as a function of the context and scientific progress. Ethical issues must be continually questioned because they reflect the infinite complexity of the living world. We consider that the living world is focused on organisms and their future – organisms that interact with each other and with their environment, compatible with an ongoing world, simultaneously complex, historical and open. Nevertheless, certain fundamental principles will always be applied, regardless of contextual and conceptual changes.
